# The effectiveness of the pregnancy adapted YEARS algorithm to safely identify patients for CT pulmonary angiogram in pregnant and puerperal patients suspected of having pulmonary embolism

**DOI:** 10.4102/sajr.v26i1.2454

**Published:** 2022-07-29

**Authors:** Riaan Potgieter, Piet Becker, Farhana Suleman

**Affiliations:** 1 Department of Diagnostic Radiology, Faculty of Health Sciences, University of Pretoria, Pretoria, South Africa; 2 Department of Statistics, Faculty of Health Sciences, University of Pretoria, Pretoria, South Africa

**Keywords:** pulmonary embolism, CT pulmonary angiogram, pregnancy-adapted YEARS algorithm, radiation safety, pregnancy, puerperium, persistent tachycardia, breast cancer

## Abstract

**Background:**

Pulmonary thromboembolism is one of the leading causes of maternal death worldwide. Globally there has been increasing physician reliance on CT pulmonary angiogram for definitive diagnoses and exclusion of pulmonary thromboembolism. The problem, however, arises when considering the high radiation penalty from performing these investigations, highlighted by the low diagnostic yield. Of recent, the pregnancy-adapted YEARS algorithm has shown promise in international studies as a possible alternative for stratifying risk of pulmonary thromboembolism during the pregnancy and puerperal period.

**Objectives:**

To determine the effectiveness of the pregnancy adapted YEARS algorithm to safely minimise the number of true negative CT pulmonary angiograms for patients suspected of having pulmonary embolism in our clinical setting.

**Method:**

A cross-sectional study was performed in a tertiary hospital in Gauteng on puerperal and pregnant patients suspected of having pulmonary embolism. We retrospectively applied the pregnancy adapted YEARS algorithm and reviewed the various outcomes.

**Results:**

The pregnancy adapted YEARS algorithm proved effective in safely identifying patients for CT pulmonary angiography. By retrospectively applying the algorithm, there could have been a 25.7% scan reduction, whilst maintaining a negative predictive value of 100.0%.

**Conclusion:**

As physician reliance on radiological investigations increases, we must remain cognisant of the added radiation exposure and the long-term adverse effects of ionising radiation. The pregnancy-adapted YEARS algorithm provides a safe, reproducible alternative to aid our bid going forward.

## Introduction

Pulmonary thromboembolism is a well-known complication during the pregnancy and puerperal periods.^1^ In the 2017 Saving Mothers Report, as well as in previous iterations, it is listed as one of the leading causes of maternal mortality in South Africa, causing roughly 30–40 maternal deaths per year.^2^ The physiological changes that take place during pregnancy render women especially vulnerable to this complication during their pregnancy and puerperal periods, with an estimated 5-fold increased risk when compared to age-related control groups.^3^ In addition, given the rising pandemic of obesity and increasing caesarean section rates in South Africa, both of which have been identified as independent risk factors, pulmonary thromboembolism in the South African context is a disease entity of justified concern.^2,4,5^

The clinical diagnosis of pulmonary thromboembolism is known to be challenging, given the fact that normal physiological changes that take place during pregnancy can mimic disease. Lower limb swelling, shortness of breath, and an increase in heart rate are all symptoms that can be experienced during the normal pregnancy and puerperal periods. Furthermore, established clinical criteria outside of pregnancy such as the Wells score, has to a large extent not been proven valid during the pregnancy and puerperal periods.^6,7^ Given this fact, there has been an increasing trend of clinicians relying on radiological methods to assist in diagnosing and/or excluding pulmonary thromboembolism and in the context of pregnancy, clinicians are over investigating suspected cases, with an exceptionally low diagnostic yield of 5% versus 15% – 20% in non-pregnant patients.^8,9^

CT pulmonary angiography is the investigation of choice in many institutions, including our own, for diagnosing pulmonary thromboembolism. It relies on contrasted imaging of the chest to opacify the pulmonary vasculature. In doing so, filling defects in the pulmonary arteries and associated complications of pulmonary thromboembolism can be detected. In addition, other unrelated pathologies (such as pneumonia or heart failure) that might explain the patient’s presenting symptoms can be diagnosed.^10^ There is however a trade-off in the form of radiation exposure to the patient. Foetal radiation exposure during CT imaging limited to the maternal chest is negligible, regardless of the use of radiation shielding.^11^ Uncertainty regarding the in-utero effects of iodinated contrast on the foetal thyroid gland has also not been correlated with significant postpartum side-effects.^12^ A factor for concern however is radiation exposure to gravid breast tissue, where it is estimated that a single CT pulmonary angiogram study increases a woman’s lifetime risk of developing breast cancer by 13.6%.^1,11^ This is a distressing figure given the fact that breast cancer is the most common cancer in women, with an increasing incidence worldwide.^13^ The use of bismuth breast shields and limited radiological techniques has been proven effective in lowering radiation dose to the female breast, although not a common practise in our institution.^14^ Ventilation/perfusion (V/Q) scans offer a more than 10 fold reduction in breast radiation dose in addition to mitigating the risk of adverse reactions from iodinated intravenous contrast.^11^ Unfortunately V/Q scans are underutilised at our institution given its unavailability in the after-hours setting. From the aforementioned information, it is evident that there is a need for a diagnostic protocol that balances the urgency of the disease with the potential adverse effects of radiation exposure. It should also consider the physiological changes and disease mimics in pregnancy.

The YEARS study was conducted in 2015-2016, and is currently under scrutiny by the international community as a proposed alternative algorithm for risk stratification in cases where pulmonary thromboembolism is suspected. It relies on disease-specific signs and symptoms, case-specific D-dimer levels and the utilisation of lower limb Doppler ultrasound to prioritise patients that need further imaging workup with CT pulmonary angiogram. The study resulted in the safe exclusion of pulmonary emboli, with a 14% reduction in required CT pulmonary angiogram studies.^15^ The YEARS algorithm has also been adapted for use in pregnancy, with one study showing a 39% reduction in CT pulmonary angiogram utilisation with the safe exclusion of pulmonary thromboembolism.^16^

## Methods

This was a cross-sectional study performed at Kalafong Provincial Tertiary Hospital Department of Radiology. In-patients presenting with suspected pulmonary embolism in the pregnancy and puerperal periods who underwent CT pulmonary angiogram at Kalafong Provincial Tertiary Hospital Radiology Department from 1 June 2017 to 1 June 2020 were considered for the study.

A list of patients who underwent CT pulmonary angiograms in the stipulated time frame was compiled from the Phillips Intellispace portal *(ISP)* study list. Corresponding electronic reports were available from March 2019 onwards on the ISP. Studies performed prior to this were accessed from the printed and filed reports at the Kalafong Department of Radiology and were physically reviewed in the department. The study list was then abbreviated to include only patients during the pregnancy and puerperal periods who met the inclusion criteria. Patient files were reviewed on site and D-dimer results were retrieved from the national health laboratory service (NHLS) online portal using the patient’s demographic information available from the patient study list.

**FIGURE 1:**
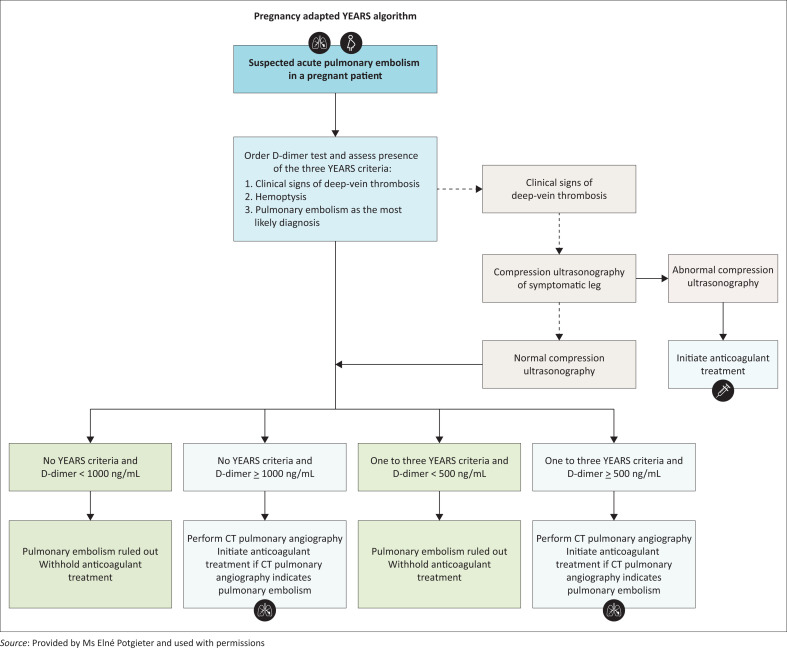
Pregnancy-adapted YEARS algorithm.

The pregnancy-adapted YEARS algorithm used to aid clinical decision making is detailed in Figure 1. The pregnancy-adapted YEARS algorithm uses a step wise approach based on clinical findings, D-dimer values and compression ultrasonography to guide clinicians as to whether further radiological workup with CT pulmonary angiogram is necessary.

A retrospective comparison was made with regard to the CTPA outcome and the pregnancy-adapted YEARS algorithm criteria. Patient demographic and clinical information was tabulated, in addition to the outcome of their CT pulmonary angiogram and D-dimer levels during hospital stay using Google Sheets.

Clinical information required included:

- Are there clinical signs of deep vein thrombosis?
- Does the patient have haemoptysis?
- Is pulmonary embolism the most likely diagnosis?

For quantitative measurement, CT report findings were assigned Boolean values:

- CT pulmonary angiogram (CTPA) positive for pulmonary embolism.
- Normal CTPA study – including incidental and non-pathological findings, that is, negative study.
- CTPA negative for pulmonary embolism but positive for other pathology likely accountable for patient’s presenting symptoms.

Patients were further categorised according to the pregnancy-adapted YEARS algorithm as either:

- CTPA was indicated.
- CTPA was not-indicated.

### Ethical considerations

The research was approved by the Ethics Review Panel of the University of Pretoria (REC #377/2021) prior to data collection. Permission was also granted by the medical manager of Kalafong Provincial Tertiary Hospital to access patient records and radiological investigations.

## Results

This descriptive study reports the findings as frequency and percentage, and most importantly, documents the sensitivity of the pregnancy-adapted YEARS algorithm.

**FIGURE 2:**
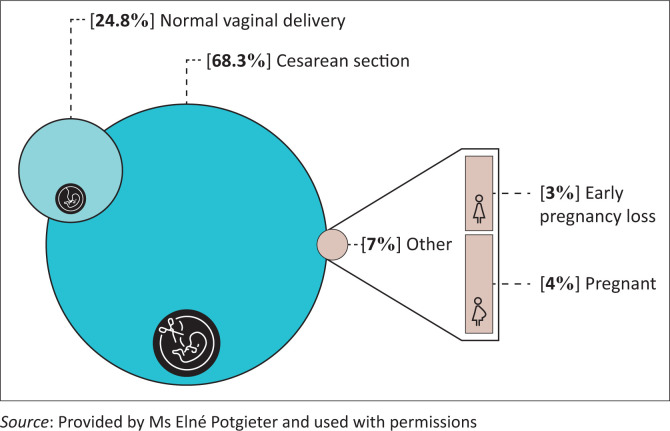
Method of delivery and timing of pregnancy (*N* = 101).

CT pulmonary angiograms were performed on a total of 131 patients during the study period. In 30 patients, the clinical information required to retrospectively compile the pregnancy-adapted YEARS algorithm was missing and these patients were excluded from the study. Missing information was attributable to D-dimer results not being performed during patient hospital stay and files missing from patient records during data collection. A total of 101 female patients during the pregnancy and puerperal periods met the inclusion criteria and were included in the study. Patient timing of pregnancy and method of delivery is presented in Figure 2.

**FIGURE 3:**
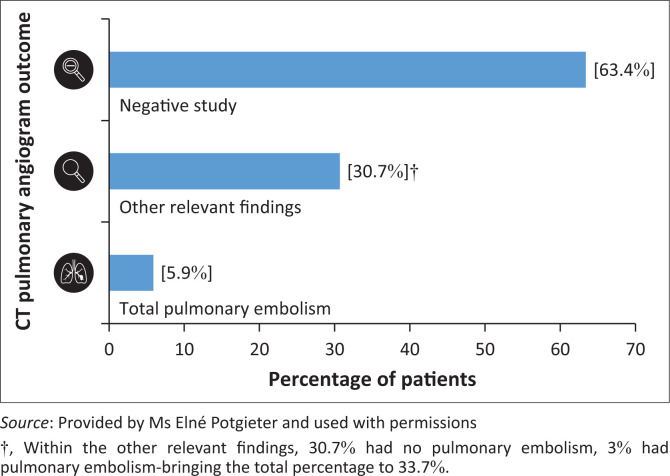
CT pulmonary angiogram outcome.

Of the 101 patients who underwent CT pulmonary angiogram, six patients (5.9%) were diagnosed with pulmonary embolism. Sixty-four studies were negative for pulmonary embolism and did not reveal any contributory findings to the patient’s diagnosis (63.4%).) (Figure 3). In 34 instances, CT pulmonary angiogram provided additional diagnostic information (33.7%). In addition, three of these studies demonstrated the presence of pulmonary embolism as well. Additional diagnostic findings yielded included puerperal sepsis (*n* = 20; 19.8%), cardiovascular disease, including dilated cardiomyopathy and heart failure (*n* = 10; 9.9%), and complications pertaining to HELLP syndrome (*n* = 2; 2.0%). In one instance, CT revealed diffuse pulmonary cystic change consistent with lymphangiomatosis (1.0%). One patient was also diagnosed with a mucous plug resulting in segmental atelectasis (1.0%). With regard to pregnancy timing, all six patients (100.0%) diagnosed with pulmonary embolism were in the puerperal period. Three patients (50.0%) delivered via caesarean section, one (16.7%) delivered via normal vaginal delivery and two suffered early pregnancy loss (33.3%), of which one (16.7%) had a miscarriage and one (16.7%) had a laparotomy for a ruptured tubal ectopic pregnancy.

**FIGURE 4:**
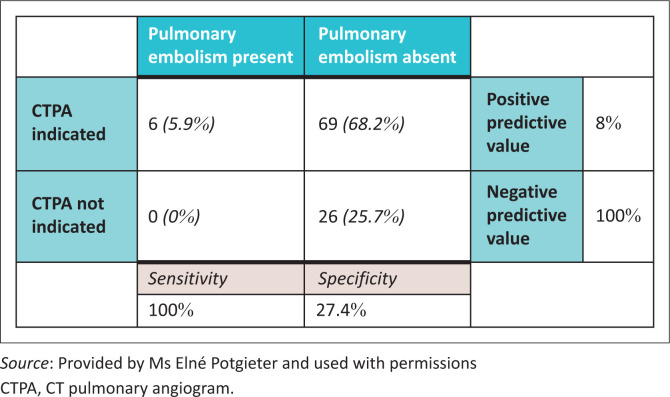
Agreement of YEARS algorithm with CTPA outcome.

When we retrospectively applied the pregnancy-adapted YEARS algorithm, it was revealed that in 26 cases (25.7%) a CT pulmonary angiogram was not indicated. Of these 26 cases, none (*n* = 0; 0.0%) of the patients were diagnosed with pulmonary embolism. The pregnancy adapted YEARS algorithm thus had a 100.0% sensitivity for identifying true negatives (100.0%; 95% CI: 54–100) Figure 4.

**FIGURE 5:**
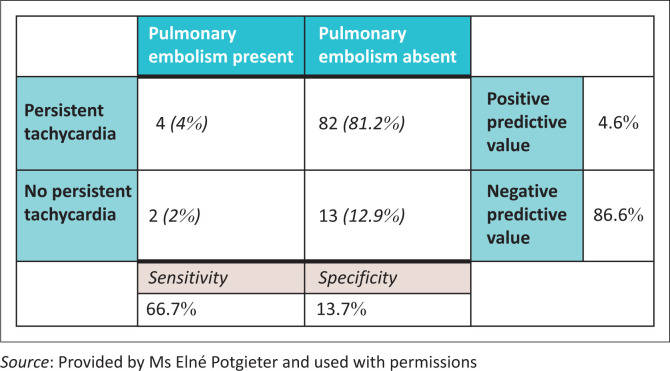
Agreement of persistent tachycardia with pulmonary embolism.

The primary indication for referral was persistent tachycardia (*n* = 86; 85.1%). In four cases (4.0%), the diagnoses of pulmonary embolism were made (Figure 5). Referral indication for the other two cases included dyspnoea and chest pain, which were the second and third most common reasons for referral, respectively. In one instance patient referral was based on persistent tachycardia in addition to acute respiratory distress.

In our study population, the most common disease entities, and final clinical diagnoses attributable to the patient presentation included puerperal sepsis in 53 instances, anaemia in 26 instances and 6 were diagnosed with having pulmonary embolism.

## Discussion

This study demonstrated the effectiveness of the pregnancy adapted YEARS algorithm to safely identify patients for CT pulmonary angiogram in pregnant and puerperal patients suspected of having pulmonary embolism. Applying the pregnancy-adapted YEARS algorithm for risk stratification before deciding on further radiological workup, would have reduced the number of CT pulmonary angiograms performed at our institution by 25.7%, without missing any true positives. This is in line with what international studies have demonstrated.^15,16,18^

The current study also highlighted the underutilisation of compression ultrasonography, which could have further reduced the number of CTPAs performed. Only two patients had lower limb ultrasound performed prior to CTPA. Furthermore, none of the patients diagnosed with pulmonary embolism had compression ultrasonography prior to CTPA. Since ultrasonography is not reliant on ionising radiation, its value as a special investigation appears undervalued in our bid to mitigate unnecessary radiation exposure to our patients.

Persistent tachycardia appears to be an unreliable indicator of pulmonary embolism specifically. This study revealed a positive predictive value of merely 4.6%, which is not unexpected as there are numerous other causes for tachycardia in the pregnancy and puerperal periods, whether it be physiological or pathological. This reiterates the validity of the pregnancy-adapted YEARS algorithm, as it is not reliant on maternal heart rate for risk stratification. In total, 86 patients presented with persistent tachycardia. In this subset of patients, puerperal sepsis, anaemia and cardiovascular disease were the most common underlying conditions. Here, the utilisation of other imaging techniques, including abdominal ultrasonography and X-ray, could also have potentially mitigated the need for CT pulmonary angiogram by assisting the diagnosis, and in so doing, greatly reduce patient radiation exposure. These imaging investigations remain invaluable given their availability and comparative low cost.

Patients in the puerperal period appear to be at an increased risk for developing pulmonary thromboembolism than during pregnancy. The six patients (100.0%) who were diagnosed with pulmonary embolism were all in the puerperal period. Furthermore, patients undergoing operative management, which included three patients who underwent caesarean section (50.0%) and one who had a laparotomy for a ruptured tubal ectopic pregnancy (16.7%), incorporated most of the patients diagnosed with pulmonary embolism (66.7%), suggesting that patients in the puerperal period requiring operative obstetric management are at an increased risk of developing pulmonary embolism. Both findings are in line with international studies.^3,17^

The major limitation of our study was our small sample size, which consisted of only 101 patients. Given that the study was performed in only one institution, our patient population was limited. Furthermore, our patient population was abbreviated given missing clinical information needed to retrospectively apply the pregnancy-adapted YEARS criteria in many instances, and misplaced patient files. This study has however replicated the findings of similar, larger studies and has arrived at the same conclusion, which further validates their findings.^15,16,18^

## Conclusion

As physician reliance on radiological investigations increases, we should remain cognisant of the added radiation exposure and the long-term adverse effects of ionising radiation. Of particular concern in pregnant and post-partum patients undergoing CT pulmonary angiogram for suspected pulmonary embolism is radiation exposure to gravid breast tissue. Breast cancer, being the most prevalent female cancer with rising incidence, is a disease entity of justified concern. We, therefore, need to put evidence-based measures in place to mitigate any additional risk that can further increase the prevalence of female breast cancer. The pregnancy-adapted YEARS algorithm provides a safe, reproducible alternative to aid our bid going forward.
